# Different Inhibitory Effects of Erythromycin and Chlortetracycline on Early Growth of *Brassica campestris* Seedlings

**DOI:** 10.3390/antibiotics10101273

**Published:** 2021-10-19

**Authors:** Mi Sun Cheong, Hyeonji Choe, Myeong Seon Jeong, Young-Eun Yoon, Hyun Suk Jung, Yong Bok Lee

**Affiliations:** 1 Institute of Agriculture and Life Science (IALS), Gyeongsang National University, Jinju 52828, Korea; mscheong@gnu.ac.kr; 2 Division of Applied Life Science (BK21 Four), Gyeongsang National University, Jinju 52828, Korea; mulberry1028@gnu.ac.kr (H.C.); yye209@gnu.ac.kr (Y.-E.Y.); 3 Department of Biochemistry, Kangwon National University, Chuncheon 24341, Korea; jms0727@kbsi.re.kr (M.S.J.); hsjung@kangwon.ac.kr (H.S.J.); 4 Chuncheon Center, Korea Basic Science Institute (KBSI), Chuncheon 24341, Korea

**Keywords:** erythromycin (Ery), chlortetracycline (CTC), *B. campestris*, chloroplast, photosynthesis, molecular marker

## Abstract

Veterinary antibiotics, including erythromycin (Ery) and chlortetracycline (CTC), are often detected in agricultural land. Although these contaminants affect plant growth and development, their effects on crops remain elusive. In this study, the effects of Ery and CTC on plant growth were investigated and compared by analyzing transcript abundance in *Brassica campestris* seedlings. Treatment with Ery and/or CTC reduced chlorophyll content in leaves and photosynthetic efficiency. Examination of the chloroplast ultrastructure revealed the presence of abnormally shaped plastids in response to Ery and CTC treatments. The antibiotics produced similar phenotypes of lower accumulation of photosynthetic genes, including *RBCL* and *LHCB1.1*. Analysis of the transcript levels revealed that Ery and CTC differentially down-regulated genes involved in the tetrapyrrole biosynthetic pathway and primary root growth. In the presence of Ery and CTC, chloroplasts were undeveloped and photosynthesis efficiency was reduced. These results suggest that both Ery and CTC individually affect gene expression and influence plant physiological activity, independently of one another.

## 1. Introduction

Plants are sessile organisms that are unable to escape unsuitable environments. Plant growth and development is retarded or inhibited by environmental stress, a process referred to as “the growth-defense trade-off” [1]. In the presence of stressful stimuli, the limited energy available is diverted from growth toward activating defense mechanisms. Plant growth and development phenotypes are stress specific, as particular stresses—for instance, exposure to low or high mineral concentrations or to other chronic or acute conditions—affect specific targets, such as below-ground or above-ground tissues [2]. Plant responses at the molecular level are complex and interconnected so as to diminish the effects of stress exposure and to enable adaptation to changing environments [2,3].

Antibiotics are therapeutic treatments that are widely used in animals as well as in humans. In particular, antibiotics are used in intensive animal husbandry to prevent the outbreak of disease [4]; however, up to 90% of ingested antibiotics are excreted in feces due to poor digestion or absorption [5]. Given that antibiotics are used approximately five times more often for veterinary purposes than in the treatment of humans [6], the direct application of animal waste produced by intensive farming to agricultural land affects the environment, as the chemical stability of antibiotics results in the accumulation of these contaminants in the soil [7,8,9]. Thus, human actions influence crop performance, including plant growth and development [8,10]. Many studies have demonstrated that various antibiotics found in soil can be absorbed through root systems and accumulate in plant tissues [9,11,12,13], and such contamination of food crops may threaten human health [14]. Nonetheless, a full understanding of how plant growth and development are affected by antibiotics in the soil—as well as the accumulation rates of antibiotics in the edible parts of food crops—remains elusive.

As antibiotics are an abiotic source of environmental stress, exposure to them may disrupt the cellular and molecular processes of plants and other living organisms, resulting in changes to morphogenic and physiological traits [12,15,16]. These effects differ between plant species and organs depending on the type and concentration of antibiotics [16]; for example, although a given concentration of sulfadiazine inhibits root and shoot elongation in wheat (*Triticum aestivum* L.), napa cabbage (*Brassica* *campestris*), and tomato (*Cyphomandra betacea*) to differing extents, the root growth of wheat demonstrated the highest sensitivity to this treatment [17].

Chlorophyll (Chl) is the most abundant photopigment found in most plant species and is crucial for light-harvesting and energy transduction in photosynthesis [18]. Although Chl is synthesized in the plastid by the tetrapyrrole biosynthetic pathway, all of the enzymes involved in this synthetic pathway are encoded by nuclear genes whose transcription is regulated by environmental cues, such as light [19,20].

Chloroplasts are discrete organelles within a plant cell [21] that are not only important for photosynthesis and the production or accumulation of various metabolites including Chl [22], but also serve as sensors of the external environment and internal signals [23]. The proper expression of chloroplast genes under stress conditions is crucial for chloroplast development and photosynthesis [21,24,25]. The arrangement of thylakoid membranes in the chloroplast results from adaptations to developmental transitions and environmental stress conditions [26].

Erythromycin (Ery) and chlortetracycline (CTC) have broad spectrum antibiotic activities [16,27] and are thus commonly used in veterinary medicine. Accumulated evidence indicates that veterinary drugs affect plant growth and development. We previously demonstrated that the treatment of *B. campestris* with Ery and CTC during the seedling growth period results in reduced Chl content [28,29]. Not surprisingly, these antibiotics were accumulated in the shoots of *B. campestris* seedlings [28,29]. Additionally, CTC accumulation in the leaves was greater during longer cultivation periods and in plants exposed to higher concentrations of antibiotics [29]. Physiologically, CTC blocks RBOH-mediated oxidative signaling in *B. campestris* and inhibits primary root growth [29], while Ery influences the accumulation of photosynthetic proteins and inhibits plant development [28]. In this study, we compared the phytotoxicity of Ery and CTC, using *B. campestris* seedlings, and investigated the combinatory effects of these antibiotics on plant growth and development, including chloroplast development.

## 2. Results

### 2.1. Erythromycin (Ery) and Chlortetracycline (CTC) Individually Affect Physiological Phenotypes

The main effects of Ery and CTC on plant physiology are a decrease in the Chl level and a reduction in primary root growth [28,29]. We therefore investigated whether the extent of the effect of a mixture of these antibiotics on plant growth and development could be described by (1) the sum of the individual antibiotic effects; (2) a synergistic effect; or (3) an antagonistic relationship. We also determined whether there was an interaction between these two antibiotics.

**Figure 1:**
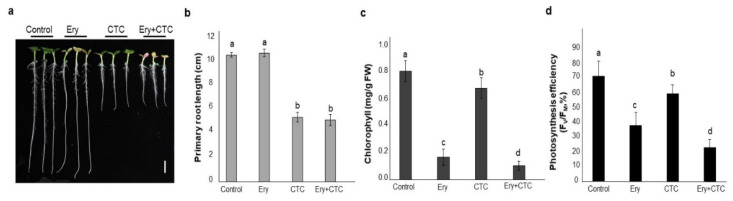
**Growth of *Brassicacampestris* seedlings treated with the veterinary antibiotics, erythromycin or chlortetracycline.** Surface-sterilized seeds were placed on agar plates in the absence (control) or presence of erythromycin (Ery), chlortetracycline (CTC), or a combination of the two (Ery + CTC) and grown vertically for 5 days. (**a**) Morphological phenotypes at day 5. (**b**) Primary root length. Primary root length was measured from photographs using ImageJ software Data are means ± SD (*n* = 39). (**c**) Total chlorophyll (Chl) content (Chl_a_+Chl_b_). Chl was extracted from detached cotyledons using methanol. Data are means ± SD (*n* = 39). (**d**) Photosynthetic efficiency (F_v_/F_m_). Data are means ± SD (*n* = 25). Photosynthetic efficiency in cotyledon leaves (*n* = 25) was measured, using a photosynthesis yield analyzer (MINI-PAM-II). Different letters above bars indicate statistically significant difference within each treatment as determined by one-way analysis of variance (ANOVA) and Tukey’s honestly significant difference (HSD) test, *p* < 0.05. All the experiments used 5 mg/L of the indicated antibiotics and were replicated four times with similar results.

We first performed seedling growth assays to compare the primary root length and Chl content of *B. campestris* seedlings grown on agar plates containing either Ery or CTC, both Ery and CTC, or no antibiotics (Figure 1). Seedlings treated with 5 mg/L Ery demonstrated a reduction in Chl content without root growth inhibition [28], whereas seedlings treated with 5 mg/L CTC demonstrated inhibited growth and development, including reductions in both Chl content and primary root length [29]. Antibiotic concentrations were established based on phenotypic observations following exposure to a single antibiotic in previous reports [28,29]. Treatment with both 5 mg/L Ery and 5 mg/L CTC resulted in root growth inhibition and lower accumulation of Chl. Although the reduction in root length following dual treatment did not differ significantly from treatment with 5 mg/L CTC alone (Figure 1b), the seedlings treated with both antibiotics had the lowest Chl levels of the four treatment groups and demonstrated a level of inhibition equivalent to the individual effects of Ery and CTC combined (Figure 1c).

Next, to further investigate whether an interaction between Ery and CTC exists at the chlorophyll level, we analyzed photosynthetic efficiency. Both Ery and CTC inhibited photosynthesis by reducing the maximum quantum yield (F_v_/F_m_) below that of the control (Figure 1d); this result was consistent with the changes seen in chlorophyll levels (Figure 1c). Combined treatment with Ery + CTC was equivalent to the sum of the inhibitory effects of the two individual treatments, indicating that, although treatment with either Ery or CTC produced similar plant growth and development phenotypes, the two antibiotics acted independently to inhibit primary root growth and reduce chlorophyll content.

To better understand how Ery and CTC inhibit photosynthesis, we examined seedling growth in the absence of light. The morphological phenotypes of dark-grown seedlings treated with antibiotics revealed that CTC inhibited primary root growth and that treatment with Ery + CTC had a similar effect on root length to treatment with CTC alone (Figure S1). These results indicated that the effect of CTC on root growth did not depend on light. Dark-grown seedlings treated with a higher concentration of Ery (10 mg/L) produced a similar morphological phenotype to that seen in the untreated control group (Figure S2d,e), despite this concentration inhibiting root growth in light-grown seedlings (Figure S2a,b) [28], which suggests that Ery interferes with reactions in a light-mediated pathway during seedling growth.

**Figure 2:**
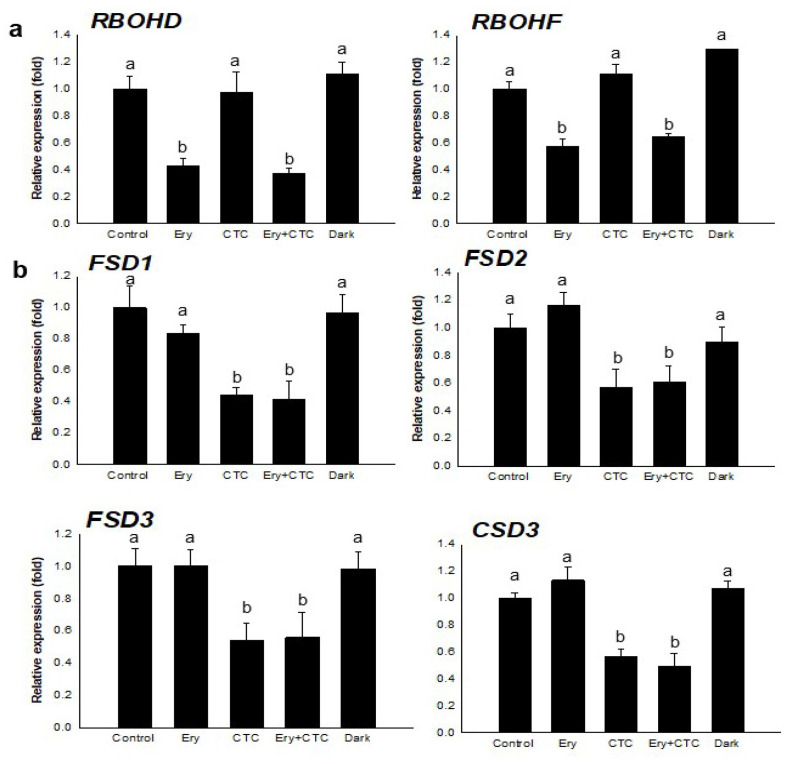
**Effect of veterinary antibiotics on expression of *RBOH* and *SOD* genes.** (**a**) Expression of *RBOHD* and *RBOHF.* (**b**) Expression of *SODs*: *FSD1*, *FSD2*, *FSD3*, and *CSD3.* The relative expression (fold change) was determined by qRT-PCR analysis of total RNA extracted from whole *B. campestris* seedlings grown for 5 days under the following conditions: control, no antibiotics; Ery, 5 mg/L erythromycin; CTC, 5 mg/L chlortetracycline; Ery + CTC, 5 mg/L erythromycin and 5 mg/L chlortetracycline; dark, no antibiotics and complete darkness for 5 days. Expression of each gene was normalized against expression of the reference gene *EF1a*. Bars represent means ± SD (*n* = 36); the experiments were replicated three times with similar results. Different letters above bars indicate a statistically significant difference within each treatment as determined by one-way analysis of variance (ANOVA) and Tukey’s honestly significant difference (HSD) test, *p* < 0.05.

CTC inhibits primary root growth by blocking an NADPH oxidase/respiratory burst oxidase homolog (RBOH)-mediated oxidative signaling pathway [29]. As CTC and Ery + CTC treatments inhibited primary root elongation (Figure 1a,b), we performed qRT-PCR to analyze expression of the oxidative marker genes *RBOHD/F* and *SOD*s, including *FSD1*, *FSD2*, *FSD3*, and *CSD3* (Figure 2). Treatment of seedlings with CTC did not affect expression of *RBOHD/F*, but expression of these genes was down-regulated in Ery-treated seedlings. Similarly, treatment with CTC resulted in down-regulation of the *SOD* genes *FSD1* and *CSD3*, but Ery treatment did not. The inhibition of root growth due to treatment with Ery + CTC thus resulted from the effect of CTC on an oxidative stress signaling pathway, with Ery acting on a different RBOH-mediated signaling pathway. Taken together, our results indicate that Ery and CTC acted on different pathways to inhibit the growth and development of *B. campestris*, as measured by their effects on Chl accumulation and primary root growth. We further suggest that the effect of Ery and CTC together is the sum of their individual effects—neither synergistic nor hostile to one another—on the growth of *B. campestris* seedlings.

### 2.2. Ery and CTC Have Different Effects on Expression of Genes in a Tetrapyrrole Biosynthetic Pathway

To understand how treatment with Ery or CTC reduced the Chl content, we analyzed the expression of genes in the tetrapyrrole biosynthetic pathway, an important part of the Chl metabolism. In higher plants, the Chl metabolism is catalyzed by a series of enzyme complexes and is regulated by three steps: Chl synthesis, the Chl cycle, and Chl degradation. These steps are commonly referred to as the tetrapyrrole biosynthetic pathway (Figure S3) [18]. Chl biosynthesis is one of the most important light-dependent cellular processes of plants and begins with the conversion of glutamic acid to protoporphyrin IX (Figure S3).

Treatment with Ery and CTC did not alter expression of the genes *GSA* (*Bra035836* and *Bra038646*), *HEMC* (*Bra007749*), *PPOX* (*HEMG1*; *Bra033317*), or *PPO* (*HEMG2*; *Bra008772*) (Figure S4a), all of which are involved in sequential steps of protoporphyrin IX biosynthesis.

**Figure 3:**
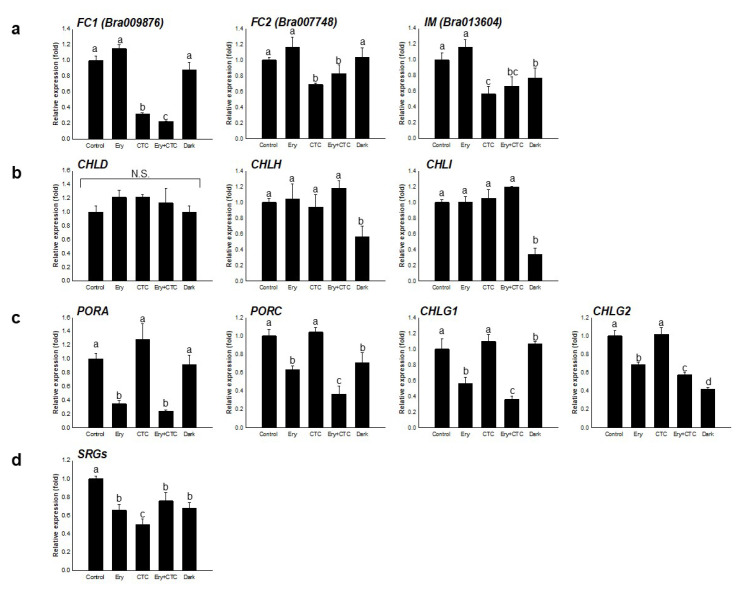
**Effect of veterinary antibiotics on expression ofgenes involved in the regulation of chlorophyll content.** Expression levels of the genes marked in bold in Figure S3 were determined by qRT-PCR analysis of total RNA extracted from *B. campestris* seedlings grown in the light for 5 days under the following conditions: control, no antibiotics; Ery, 5 mg/L erythromycin; CTC, 5 mg/L chlortetracycline; Ery + CTC, 5 mg/L erythromycin and 5 mg/L chlortetracycline; dark, no antibiotics and complete darkness for 5 days. (**a**) Expression of *FC1* (*Bra009876*), *FC2* (*Bra007748*), and *IMMUTANS* (*IM*; *Bra013604*). (**b**) Expression of *CHLH* (*Bra006208*), *CHLD* (*Bra018619*), and *CHLI* (*Bra012595* and *Bra013314*). (**c**) Expression of *PORA* (*Bra003004*), *PORC* (*Bra033415* and *Bra030540*), *CHLG1* (*Bra040893*), and *CHLG2* (*Bra028411*). (**d**) Expression of *SGRs* (*Bra019346* and *Bra000755*). Expression of each gene was normalized against expression of the reference gene *EF1α*. Bars represent means ± SD (*n* = 20); the experiments were replicated three times with similar results. Different letters above bars indicate a statistically significant difference within each treatment as determined by one-way analysis of variance (ANOVA) and Tukey’s honestly significant difference (HSD) test, *p* < 0.05. N.S. = no significant differences among treatments.

In the next step of the Chl biosynthesis pathway, protoporphyrin IX oxidation, protoporphyrin IX enters two different pathways that synthesize heme and Mg-protoporphyrin IX via the catalytic activities of ferrochelatase (FC, ferroxidases) and magnesium chelatase (CHLH/D/I), respectively. Treatment with either Ery or CTC had different effects on the expression of these genes; more specifically, the ferroxidases *FC1* (*Bra009876*) and *FC2* (*Bra007748)* were down-regulated following treatment with CTC, whereas treatment with Ery did not alter expression of either of these oxidases, or that of the magnesium chelatases, *CHLH* (*Bra006208*), *CHLD* (*Bra018619*), and *CHLI* (*Bra012595* and *Bra013314*) (Figure 3a,b). To confirm the expression of oxidase genes during chlorophyll biosynthesis, we additionally tested *IMMUTANS* (*IM,* also known as *PLASTID TERMINAL OXIDASE*, *PTOX;* *Bra013604*), an alternative oxidase that functions in the chloroplast [30]. As with ferroxidase, expression of *IM* was reduced following treatment with CTC and growth in the dark, but not following exposure to Ery (Figure 3a). Thus, CTC treatment led to down-regulation of genes involved in heme biosynthesis, which is important for chloroplast development.

We also investigated subsequent steps in the chlorophyll biosynthetic pathway. Growth in darkness or treatment with Ery suppressed the expression of *PORA* (*Bra003004*) and *PORC* (*Bra033415* and *Bra030540*), which are genes that encode NADPH- and light-dependent oxidase protochlorophyllide oxidoreductase (POR), which plays a rate-limiting role in Chl biosynthesis [18] (Figure 3c). The *B. campestris CHLOROPHYLL SYNTHASE* (*CHLG*) genes encoding Chl synthase are annotated as two different isoforms, *CHLG1* (*Bra040893*) and *CHLG2* (*Bra028411*); levels of both transcripts decreased following treatment with Ery (Figure 3c). Ery thus appeared to reduce the expression of chlorophyll biosynthetic genes encoding enzymes that require light for their activity, suggesting that down-regulation of the chlorophyll biosynthesis pathway and reduced chlorophyll content in Ery-treated seedlings resulted from the decreased activity of Chl synthase.

**Figure 4:**
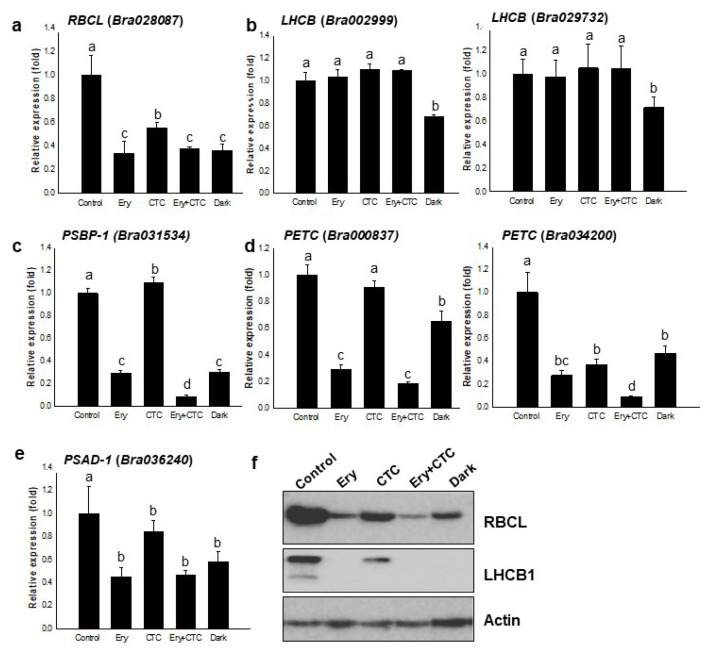
**Effect of veterinary antibiotics onexpression of genes and proteins involved in photosynthesis.** (**a**) Expression of *RBCL* (ribulose bisphosphate carboxylase large subunit; *Bra028087*). (**b**) Expression of *LHCB* (light-harvesting complex Chl a/b-binding protein; *Bra002999* and *Bra029732)*. (**c**) Expression of *PSBP-1* (photosynthetic II subunit P-1; *Bra031534*). (**d**) Expression of *PETC* (photosynthetic electron transfer C; *Bra000837* and *Bra034200*). (**e**) Expression of *PSAD-1* (photosystem I subunit D-1; *Bra036240*). The relative expression (fold change) was determined by qRT-PCR analysis of total RNA extracted from *B. campestris* seedlings grown in the light for 5 days under the following conditions: control, no antibiotics; Ery, 5 mg/L erythromycin; CTC, 5 mg/L chlortetracycline; Ery + CTC, 5 mg/L erythromycin and 5 mg/L chlortetracycline; dark, no antibiotics and complete darkness for 5 days. Expression of each gene was normalized against expression of the reference gene *EF1α*. Bars represent means ± SD (*n* = 24); the experiments were replicated four times with similar results. Different letters above bars indicate statistically significant difference within each treatment as determined by one-way analysis of variance (ANOVA) and Tukey’s honestly significant difference (HSD) test, *p* < 0.05. (**f**) Abundance of RBCL, LHCB1, and actin proteins. Immunoblot analysis using anti-RBCL, anti-LHCB1, and anti-actin antibodies of total protein extracted from seedlings grown as in Figure 4a–e; the anti-actin immunoblot was used as a loading control.

We next analyzed the transcript levels of *CAO* (*Bra036948*), *NYC* (*Bra034882* and *Bra032697*), and *NOL* (*Bra005827*). These genes act in the Chl cycle that interconverts Chl a and Chl b [21]. Treatment with Ery and CTC did not affect the expression of genes involved in the Chl cycle (Figure S4b), which suggests that Ery and CTC did not affect the Chl a to Chl b ratio. This finding is consistent with previous reports that, in *B. campestris*, this ratio is unaltered by treatment with Ery or CTC, despite a decrease in total Chl content [28,29]. Treatment with both antibiotics decreased the transcript abundance of Chl degradation genes, as expression of the *STAY-GREEN* (*SGR*) genes *SGR1* (*Bra019346*) and *SGR2* (*Bra000755*), which act in the Chl degradation pathway, was inhibited by treatment with Ery and CTC (Figure 4d). These genes are important for Chl degradation [31,32]. Together, these results indicate that Ery and CTC affected the expression of genes involved in the tetrapyrrole biosynthetic pathway and suggest that these antibiotics acted in different ways to influence the molecular regulation of Chl accumulation.

We further analyzed the transcription of other genes affecting Chl accumulation in plants in addition to those acting in the tetrapyrrole biosynthetic pathway. *SNOWY COTYLEDON 1* (SCO1) encodes a chloroplast-localized elongation factor G that plays a role in chloroplast development in cotyledons in Arabidopsis (*Arabidopsis thaliana*), but not in the Chl biosynthetic pathway [33]; the knock-out mutant of this gene has pale cotyledons. Additionally, an analysis to identify differentially expressed proteins (DEPs) in the chlorophyll-deficient mutant (*cdm*) of *B.* *campestris* and wild-type plants found that many ribosomal proteins, such as RPL, were up-regulated in *cdm*; this difference in protein abundance was consistent with a qRT-PCR analysis of gene expression [34]. As transcript abundance of these genes was related to a Chl-deficient phenotype [33,34], we used qRT-PCR to analyze expression of *SCO1* (*Bra027021*) and *RPL21* (*Bra036731*) in *B.* *campestris*. Both of these genes are encoded in the nuclear genome. Their expression was unchanged by treatments with Ery, CTC, Ery + CTC, or darkness (Figure S5), suggesting that these antibiotics did not act on a chlorophyll accumulation pathway mediated by SCO1 and/or RPL21.

### 2.3. Ery and CTC Affect Expression of the Photosynthetic Apparatus

To observe in more detail the effects of Ery and CTC that result from down-regulation of the tetrapyrrole biosynthetic pathway in *B.* *campestris*, we employed qRT-PCR to measure the expression levels of photosynthesis-related genes and compared the effects of Ery and CTC (Figure 4). Candidate genes were selected from two different proteomic studies: one, by Yoon et al. [28], was a group of differentially abundant proteins (DAPs) identified as functioning in photosynthesis, while the other was a list produced by Lee [35] of the most abundant proteins in *Brassica rapa* seedling leaves. As photosynthesis is a light-dependent biochemical reaction [36], we compared the effects of antibiotic treatment and darkness on the expression of photosynthetic genes. *Bra028087* is a gene from the chloroplast genome that encodes ribulose bisphosphate carboxylase (RBCL); its expression was reduced by treatment with Ery and CTC (Figure 4a). Of the many isoforms of light-harvesting complex proteins and Chl a/b-binding proteins, we randomly selected *Bra0029999* and *Bra029732* encoding LHCB1. Expression of these two genes was not altered by treatment with Ery and CTC, although it was inhibited by darkness (Figure 4b).

We next examined expression of *Bra031534*, *Bra000837*, *Bra034200*, and *Bra036240*, which are genes involved in the light reaction of photosynthesis. *Bra031534* is a nuclear gene encoding photosystem II subunit P-1 (PSBP-1); its expression was reduced by Ery treatment and by darkness (Figure 4c). The expression of *Bra000837* and *Bra034200* encoding PETC, which functions in transferring an electron from the cytochrome b6/f complex photosystem II, was also inhibited by Ery and darkness (Figure 4d). Interestingly, CTC treatment did not suppress expression of *Bra000837* but did suppress *Bra034200* expression (Figure 4d). The expression of *Bra036240*, which encodes photosystem I subunit D-1, was suppressed by Ery and by darkness, but not by CTC (Figure 4e); these results resembled those obtained for *Bra000837*. Treatments with Ery and CTC thus produced a similar effect to darkness on the expression of photosystem reaction components.

To further confirm the photosynthetic components, we performed immunoblot analyses of the crude protein extracts of Ery- and/or CTC-treated seedlings with anti-RBCL, anti-LHCB1, and anti-actin (Figure 4f). As expected, antibiotic treatment dramatically decreased RBCL abundance (Figure 4f) [28]. LHCB1 abundance was significantly reduced by treatment with Ery and/or CTC, as well as by darkness (Figure 4f). While we found that treatment with Ery and/or CTC did not alter the transcript levels of a *B. campestris* gene encoding LHCB—although dark treatment did (Figure 4b)—antibiotic treatments did decrease the protein abundance to a similar extent as darkness (Figure 4f). This finding indicates that Ery and CTC acted at the transcriptional and translational levels to down-regulate photosynthetic components and supports the hypothesis that these antibiotics inhibit photosynthesis efficiency.

### 2.4. Ery and CTC Inhibit Chloroplast Formation at the Ultrastructural Level

**Figure 5:**
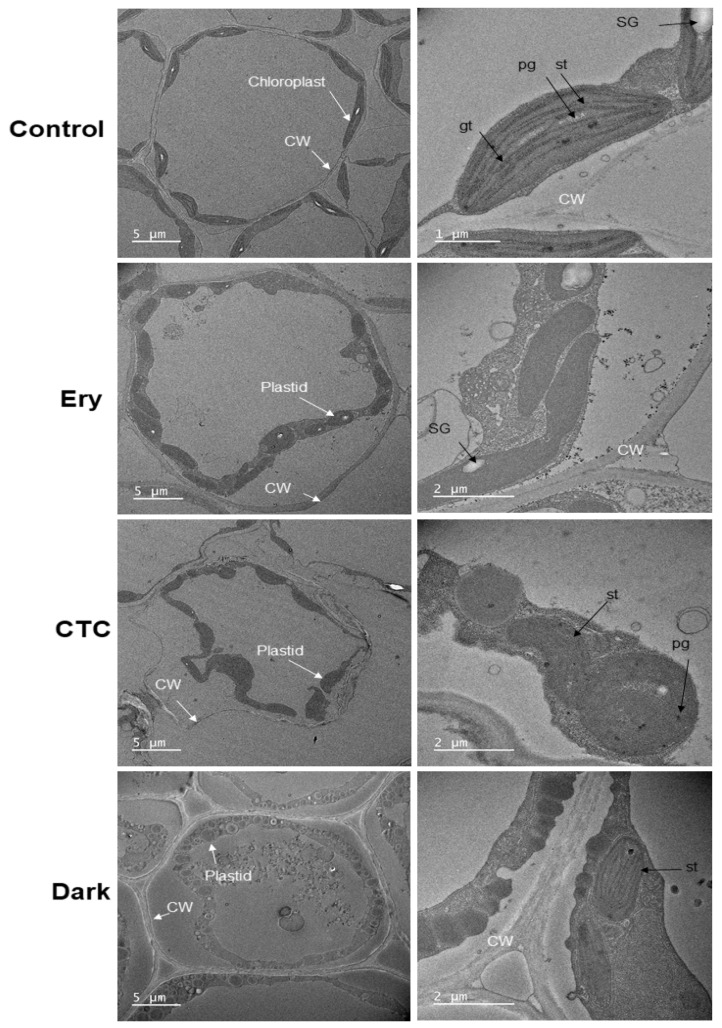
**Ultrastructural changes in epidermal plastids.** Transmission electron microscopy of the plastids was performed using cotyledons of 5-day-old seedlings grown under the following conditions: control, no antibiotics; Ery, 5 mg/L erythromycin; CTC, 5 mg/L chlortetracycline; dark, no antibiotics and darkness for 5 days. Plastids in control seedlings contained thylakoid membranes (th), unlike those in seedlings treated with Ery, CTC, or darkness. St, stroma thylakoid membrane; gt, granal thylakoid membrane; CW, cell wall; Vc, vacuole-like structure; PG, plastoglobuli; SG, starch granule.

The chloroplast is a plant organelle essential for photosynthesis [21]. The proper regulation of genes that play a role in the chloroplast is crucial for chloroplast development and photosynthesis. We used transmission electron microscopy to examine the ultrastructure of chloroplasts in light-grown seedlings treated with Ery and CTC and compared this with the undeveloped plastids of dark-grown seedlings (Figure 5). Chloroplasts in control cotyledons demonstrated a well-matured ultrastructure. They contained few internal plastoglobules (PGs), stacked thylakoid membranes, and retained starch granules (Figure 5, control). By contrast, the cotyledons from dark-grown cotyledons contained undeveloped plastids with few thylakoid membranes and many PGs with a vacuole-like structure (Figure 5, dark). Plastids in the cotyledons of seedlings treated with Ery and CTC that were exposed to the same light conditions as the controls demonstrated an intermediate structure (Figure 5, Ery and CTC). These plastids contained more PGs and fewer stacked membranes than plastids in control seedlings, but a more organized structure than plastids from dark-grown seedlings. Thus, Ery and CTC inhibited plastid development and prevented chloroplasts from forming a normal ultrastructure.

## 3. Discussion

Veterinary antibiotics, such as Ery and CTC, were originally intended to prevent and treat animal diseases [4] but are frequently detected in agricultural land [37]. These substances have very stable chemical structures and thus accumulate as environmental pollutants in the soil, where their residual activity may affect ecosystems by disrupting plant growth and development [4,5,38]. Treatment of seedlings with CTC and Ery inhibits specific developmental events, such as germination [28,29]. These antibiotics were detected at higher levels in the shoots of *B. campestris* plants as a result of longer periods of cultivation or higher concentrations in the growth media [28,29], similar to previous studies, which investigated the translocation and accumulation of other antibiotics from soil to plant tissue [11,12]. Despite the fact that these antibiotics can pass along the food chain from crop plants to humans, thereby influencing human health [14,38], neither their precise physiological effect on crop plants nor the molecular basis of their phytotoxicity are fully understood.

Dual treatment with Ery and CTC indicated an additive effect on *B. campestris* seedling morphology, as these antibiotics reduced Chl accumulation and inhibited growth and development (Figure 1, Figures S1 and S2). Molecular analysis by qRT-PCR of the phytotoxic effects of Ery and CTC indicated that the antibiotics exerted their influence independently, as the changes in gene expression induced by both antibiotics revealed neither a synergistic nor an antagonistic relationship. Although both antibiotics altered the course of the growth and development of *B. campestris* seedlings by acting on an oxidative signaling pathway, they did so via different inhibitory modules. CTC inhibited primary root growth by interfering with the expression of SODs, which detoxify superoxides to hydrogen peroxide (H_2_O_2_) and down-regulate H_2_O_2_-mediated signaling at the root tip [29] (Figure 2b). Growth inhibition in seedlings treated with CTC resulted from changes in *SOD* gene expression. Interestingly, the Arabidopsis FeSOD T-DNA knockout mutants *fsd2* and *fsd3* demonstrated delayed growth, pale green leaves, and abnormal chloroplasts [39]. Similarly, the low expression of *FSDs* (Figure 2) following CTC treatment may reduce chlorophyll accumulation (Figure 1 and Figure 2). Ery also reduced chlorophyll levels to a similar extent as CTC did (Figure 1 and Figure S1). Seedlings treated with either or both antibiotics expressed lower levels of photosynthesis-related proteins (Figure 4f) and demonstrated lower photosynthetic efficiency (Figure 1d). In addition, Ery and CTC acted on the chlorophyll biosynthetic and heme formation pathways, respectively, at stages downstream from protoporphyrin IX (Figure 3 and Figure S3).

The inhibition of growth and development in response to Ery was somewhat light specific (Figure S2). Several PROTOCHLOROPHYLLIDE VINYL REDUCTASE (POR) and CHLOROPHYLL SYNTHASE (CHLG) proteins have been annotated in the *B. campestris* genome (http://brassicadb.org/brad/). PORA and PORC are nuclear-encoded enzymes that catalyze light-sensitive NADP(H)- or NAD(H)-dependent reactions and have no catalytic activity in the dark [40]. Thus, POR proteins play a rate-limiting role in Chl photosynthesis [18], as do CHLGs, which catalyze the final step in chlorophyll biosynthesis to produce Chl a [41]. Although POR expression and activity are regulated by light [18] and, in Arabidopsis, the *CHLG* transcript is only detected in green and greening tissues [41], treatment with Ery resulted in the down-regulation of *POR* and *CHLG* expression, but treatment with CTC did not (Figure 4c), suggesting that Ery inhibited expression of light-responsive genes involved in Chl biosynthesis. By contrast, treatment with CTC, but not Ery, reduced expression of *FC1* and *FC2*—genes encoding single subunit ferrochelatase enzymes that are involved in synthesizing heme—and *IM*, which encodes an enzyme that plays an important role in chloroplast differentiation (Figure 4a) [30,42] and heme biosynthesis; these enzymes act in different branches of the tetrapyrrole metabolic pathway (Figure S3) [42]. Although heme and Chl are essential components of the photosynthetic electron transfer chain, they do not always follow the same accumulation profile [43]. Thus, Ery and CTC are likely to act on different regulatory modules; however, as treatment with either results in lower Chl accumulation (Figure 1), it remains unclear whether they have the same molecular targets [28,29].

Treatment with Ery and/or CTC did not alter the expression of *CAO*, *NYL*, or *NOL*, which are genes involved in the Chl cycle (Figure S4b); this result is consistent with the observation that treatment with Ery and/or CTC did not alter the Chl a to Chl b ratio from the control value (data not shown) [28,29]. The expression of *SGR* genes, which act in the chlorophyll degradation pathway, was down-regulated following treatment with Ery and/or CTC (Figure 4d). We speculate that this may decrease Chl breakdown and maintain Chl levels by activating an unknown mechanism. In addition, CTC is known to interact with cations, such as Mg^2+^ and Ca^2+^ [44], and thus may contribute to Chl breakdown via the dechelation of Mg^2+^ in the Chl molecule [31,45]. It is therefore possible that CTC itself is involved in Chl breakdown, thereby directly reducing Chl content [46].

An analysis of the leaf proteome of *B. campestris* seedlings indicated that the most abundant proteins were Rubisco and ribosomal proteins, which accounted for 11.56% and 8.47% of total proteins, respectively [35]. Treatment with Ery reduced the expression of the photosynthetic apparatus [28], and CTC treatment had similar effects on Rubisco content and photosynthetic gene expression (Figure 5), suggesting that Ery and/or CTC affects photosynthesis efficiency (Figure 1d). Light-harvesting Chl-binding (LHC) proteins are highly abundant in *B. campestris* leaves; treatment with Ery and/or CTC did not alter expression of *Bra002999* and *Bra029732*, although these treatments dramatically reduced the Chl content of seedlings (Figure 1 and Figure 5). Chl b stabilizes the photosynthetic protein complex that contains LHC proteins, and Chl b levels determine LHC construction, which is important for photosynthesis [47]. The application of Ery and/or CTC altered LHCB levels and produced consistent effects on photosynthetic efficiency (F_v_/F_m_) (Figure 1 and Figure 4), although it remains unclear whether the antibiotics reduced LHCB levels by reducing Chl content or by regulating translation. Plastid factors, such as LHC and RBCL, control photosynthesis or chloroplast development [48]; we found that treatment with Ery and/or CTC decreased the accumulation of Rubisco, LHCB, and photosystems I and II (Figure 4) [28], suggesting that they inhibit chloroplast development, which requires not only chlorophyll accumulation, but also the proper regulation of chloroplast proteins, such as RBCL.

The development of the mature form of the chloroplast, the photosynthetic organelle, requires changes in the composition and structure of plastids [48]. PGs are thylakoid-associated droplets composed of lipids and proteins that are found in all types of plastids. Their size varies depending on abiotic stresses and developmental transitions [49]. Plastids in dark-grown seedlings contained many PGs (Figure 5, dark) to provide a readily available pool of enzymatic substrates, including for PORs, and to allow immediate Chl biosynthesis in response to a light stimulus [48,50,51,52], leading to greening and chloroplast development (Figure 5, Control) [48,52]. Chloroplasts develop a complex internal membrane system, the thylakoids, during their differentiation from plastids. This system responds and adapts to changing environmental conditions and developmental cues, although the process of thylakoid membrane biogenesis remains unknown [48,53]. The plastids in seedlings treated with Ery and/or CTC exhibited a similar shape to those seen in dark-grown seedlings and contained many vacuole-like structures, including PGs and less developed thylakoid membranes. The seedlings in the control, light-grown group formed fully developed chloroplasts with well-organized thylakoid membranes (Figure 5), suggesting that Ery and CTC blocked the ultrastructural development of chloroplast features, such as the thylakoid membrane.

In the chloroplast, SOD plays an important role in the detoxification of superoxide (O_2_^−^), which is produced during photorespiration. FSD genes originated in the plastid genome and moved to the nuclear genome by endosymbiosis during evolution [54]. Arabidopsis FSD1, FSD2, and FSD3 are targeted to stroma, thylakoid membranes, and nucleoid in chloroplasts, respectively, and a heterocomplex of FSD2 and FSD3 in the chloroplast protects chloroplast nucleoid from damage by superoxide anions and helps the chloroplast gene expression machinery [39]. Therefore, it is suggested that CTC inhibits aspects of chloroplast development, such as thylakoid membrane development, by reducing the expression of *FSDs*. It is well known that FSDs are critical for protection against oxidative stress during photorespiration as well as for chloroplast and thylakoid membrane development; however, their regulatory mechanism has not been reported [39,55].

**Figure 6:**
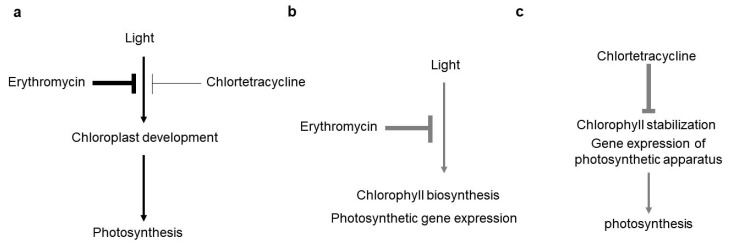
**A working model of the effects of Ery and CTC on chloroplast (Chl) development.** (**a**) Ery and CTC inhibit chloroplast development reducing the efficiency of photosynthesis. (**b**) Ery inhibits light-mediated Chl biosynthesis and the expression of photosynthetic gene expression. (**c**) CTC inhibits the stability of the photosynthetic apparatus stability, reducing the efficiency of photosynthesis. Arrows represent promoters of biological processes and T-shaped symbols represent inhibitors. Gray lines (in **b**,**c**) are known from previous reports; black lines in (**a**) are confirmed by this study.

We produced a working model summarizing all of our results (Figure 6). This figure indicates the phenotypes produced by treatment with Ery and CTC; these phenotypes are physiologically similar but arise from interference in different molecular regulatory modules acting in independent pathways (Figure 6). Treatment with both Ery and CTC reduced chlorophyll accumulation and the expression of genes involved in the photosynthetic apparatus, thus decreasing photosynthetic efficiency. Antibiotic treatment caused other aspects of chloroplast development to resemble that seen in chloroplast-defective mutants [56,57], including a decrease in well-organized thylakoid membranes and the production of many vacuole-like structures. Ery, however, acted by interfering with the expression of genes involved in a light-dependent step of the chlorophyll biosynthesis pathway, whereas CTC affected the ferroxidase pathway in heme biosynthesis.

We investigated and compared the molecular responses of *B. campestris* seedlings to the veterinary drugs Ery and CTC. Although these antibiotics produced similar physiological phenotypes—such as reduced chlorophyll contents, low photosynthesis efficiency, and chloroplast development—we found that Ery down-regulated the expression of chlorophyll biosynthetic genes, while CTC down-regulated those involved in heme production, including oxidases and SODs. These results suggest that Ery and CTC individually affect gene expression and influence plant physiological activity independently of one another during seedling growth.

## 4. Materials and Methods

### 4.1. Plant Growth for Chemical Treatments and the Measurement of Physiological Parameters

Seeds of *Brassica campestris* L. ssp. *perkinensis Rupr* were purchased from the Asia Seed Company (Seoul, Republic of Korea). For seedling growth assays, approximately 50 seeds were sterilized and placed on plates containing 1.2% agar supplemented with Ery (5 mg/L; TCI Development, Shanghai, China) and/or CTC (5 mg/L; Sigma-Aldrich, Rehovot, Israel). The seeds were incubated vertically for 4 to 5 days in a growth chamber maintained at 22 °C under continuous darkness or long-day photoperiods (16 h light/8 h dark; light intensity, 120 μEm^−2^S^−1^).

Seedlings were photographed to record growth. Primary root length was measured from photographs, using ImageJ software (http://imagej.nih.gov/ij/download.html), NIH, Bethesda, MD, USA). Chl was extracted from detached cotyledons, using methanol; the total Chl content was calculated as follows: Chla + b = 22.12A_652.0_ + 2.71A_655.2_; where A = absorbance.

To measure photosynthetic efficiency (F_v_/F_m_), a cotyledon leaf was clipped, placed in the dark for 30 min, and analyzed, using a photosynthesis yield analyzer (MINI-PAM-II; Heinz Walz GmbH, Effeltrich, Germany).

### 4.2. Total Protein Extraction and Western Blot Analysis

Total proteins were extracted from harvested seedlings. Seedlings were ground into fine powder using liquid nitrogen; two volumes of ice-chilled protein extraction buffer (1× PBS, pH 7.4, 0.1% Triton X-100) and protease inhibitor cocktail tablets (Complete Mini, Roche, Indianapolis, IN, USA) were added and the suspension was mixed well. The samples were incubated on ice for 20 min and then centrifuged for 10 min at 4 °C. The supernatant (total protein extracts) was transferred to a new sample tube.

For one-dimensional SDS-PAGE, a 20 μg aliquot of total protein extract was added to denaturing loading sample buffer (0.5 M Tris-HCl, pH 6.8, 10% SDS, 20% glycerol, 1% bromophenol blue, and 0.2% DTT) and heated at 95 °C for 5 min. The samples were subjected to SDS-PAGE and then transferred onto polyvinylidene difluoride (PVDF) membranes. The membranes were incubated with 5% nonfat dried milk in TBS-T (10 mM Tris-Cl, pH 7.4, 150 mM NaCl, and 0.1% Tween-20) for 1 h before a further incubation with antibodies against RBCL (1:5000; Agrisera, Vannas, Sweden), LHCB (1:2000; Agrisera, Vannas, Sweden), or actin (1:1000; Abcam, Seoul, Republic of Korea) for 1 h. Membranes were washed three times for 10 min. For secondary antibody treatment, membranes were incubated with a 1:3000 dilution of anti-rabbit antibody conjugated with horseradish peroxidase (HRP) for 30 min. Blots were washed with TBS-T three times for 10 min and developed, using the enhanced chemiluminescence (ECL) system.

### 4.3. Total RNA Extraction and qRT-PCR

Total RNA was extracted from 5-day-old seedlings using TRIzol reagent (Thermo Fisher Scientific, Waltham, MA, USA), according to the manufacturer’s instructions. First-strand cDNA was synthesized from 1 μg total RNA using a cDNA synthesis kit (Thermo Fisher Scientific, Waltham, MA, USA). To rescue chloroplast-expressed RNA, cDNA was synthesized, using oligo dT and random primers. The cDNA template was analyzed by qRT-PCR using a CFX Connect Real-Time PCR Detection System (Bio-Rad, Hercules, CA, USA), gene-specific primers (Supplementary Table S1), and AccuPower 2× GreenStar qPCR Master Mix (Bioneer, Daejeon, Republic of Korea). *EF1α* was used as an internal reference gene for data normalization. The qRT-PCR was performed using the following conditions: 95 °C for 5 min, followed by 45 cycles of 95 °C for 20 s, 58 °C for 20 s, and 72 °C for 20 s. The mean level of expression of each gene was determined, using the comparative Ct method (2^−ΔΔCt^). Data and error bars are indicated as means ± standard deviation (SD). The experiments were performed independently three or four times.

### 4.4. Transmission Electron Microscopy (TEM)

The samples were fixed with 2% glutaraldehyde and 2% paraformaldehyde in phosphate buffer (pH 7.4) for 1 h at 4 °C and postfixed with osmium tetroxide for 40 min at 4 °C. The samples were then dehydrated in a graded ethanol series, treated with a graded propylene oxide series, and embedded into Epon (TED Pella, Redding, CA, USA). The embedded samples were cut into ultra-thin (80 nm) sections, placed on a copper grid before being stained with uranyl acetate and lead citrate, and were observed using a transmission electron microscope (JEM-2100F, Tokyo, Japan) at 200 KV.

### 4.5. Statistical Analyses

All statistical data analyses were carried out using Minitab 18 (Minitab Inc., State College, PA, USA), with one-way analysis of variance (ANOVA) and Tukey’s honestly significant difference (HSD) test employed to identify differences, *p* < 0.05.

## Data Availability

The data that support the findings of this study are available from the corresponding author upon reasonable request.

## Supplementary Materials

The following are available online at https://www.mdpi.com/article/10.3390/antibiotics10101273/s1, Figure S1. The effect of veterinary antibiotics on growth of Brassica campestris seedlings in the dark; Figure S2. Comparison of the effects of different concentrations of Ery on Brassica campestris seedlings grown in long-day photoperiods and darkness; Figure S3. The chlorophyll biosynthesis pathway and the chlorophyll cycle; Figure S4. Effect of veterinary antibiotics on expression of genes involved in the regulation of chlorophyll content; Figure S5. Effect of veterinary antibiotics on expression of genes such as SCO1 and RPL21; Table S1. Primer sequences used in this study.
