# Supplementary material for: Different Inhibitory Effects of Erythromycin and Chlortetracycline on Early Growth of Brassica campestris Seedlings
Source: Antibiotics (Basel). 2021 Oct 19;10(10):1273. doi: 10.3390/antibiotics10101273 (PMC8532913; doi:10.3390/antibiotics10101273)
Supplement: Supplementary file 1 [file antibiotics-10-01273-s001.zip › antibiotics-1396098-supplementary.pdf]

Supporting information

Different Inhibitory Effects of Erythromycin and Chlortetracycline on Early Growth of *Brassica campestris* Seedlings

Mi Sun Cheong [1,¶], Hyeonji Choe [2,¶], Myeong Seon Jeong [3,4], Young-Eun Yoon [2], Hyun Suk Jung [3], and Yong Bok Lee [1,2,*]

1 Institute of Agriculture and Life Science (IALS), Gyeongsang National University, Jinju 52828, Republic of Korea; mscheong@gnu.ac.kr (M.S.C.)
2 Division of Applied Life Science (BK21 Four), Gyeongsang National University, Jinju 52828, Republic of Korea; mulberry1028@gnu.ac.kr (H.C.), yye209@gnu.ac.kr (Y.-E.Y.)
3 Department of Biochemistry, Kangwon National University, Chuncheon, 24341, Republic of Korea; hsjung@kangwon.ac.kr (H.S.J.)
4 Chuncheon Center, Korea Basic Science Institute (KBSI), Chuncheon 24341, Republic of Korea; jms0727@kbsi.re.kr (M.S.J.)
* Correspondence: yblee@gnu.ac.kr (Y.B.L.); Tel.: +82-55-772-1967
¶ These authors contributed equally to this study

Supplementary Materials

Figure S1. The effect of veterinary antibiotics, Ery and CTC, on growth of *Brassica campestris* seedlings in the dark.

Figure S2. Comparison of the effects of different concentrations of Ery on *Brassica campestris* seedlings grown in long-day photoperiods and darkness.

Figure S3. The chlorophyll biosynthesis pathway and the chlorophyll cycle.

Figure S4. Effect of veterinary antibiotics on expression of genes involved in the regulation of chlorophyll content.

Figure S5. Effect of veterinary antibiotics on expression of *genes such as SCO1*, and *RPL21*.

Table S1. List of primer sequences used in this study.

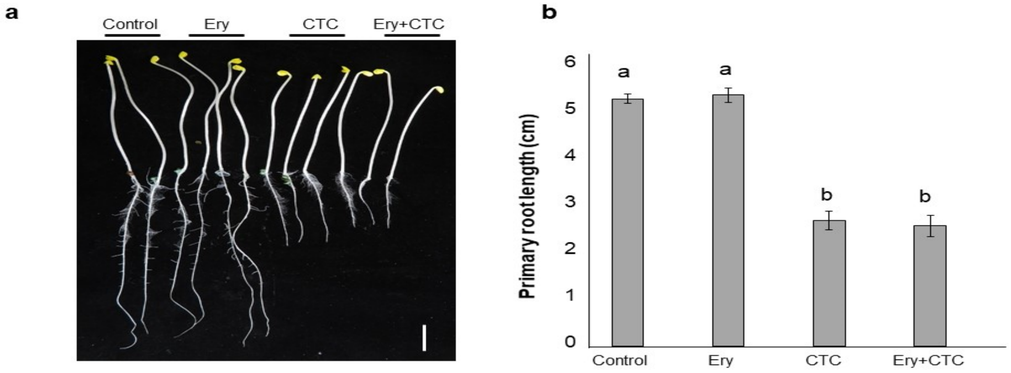

Figure S1. The effect of veterinary antibiotics, Ery and CTC, on growth of *Brassica campestris* seedlings in the dark. Sterilized seeds were placed on Ery, CTC, or Ery/CTC-containing agar medium and grown vertically under continuous dark for five days. (**a**) Morphological phenotype at day 5. White bar=1 cm (**b**) Primary root length. Data represent mean ± SD (*n* = 30). All experiments were replicated three times with similar results by using 5 mg/L indicated veterinary antibiotics. Different letters above bars indicate statistically significant difference within each treatment as determined by one-way analysis of variance (ANOVA) and Tukey's honestly significant difference (HSD), p<0.05.

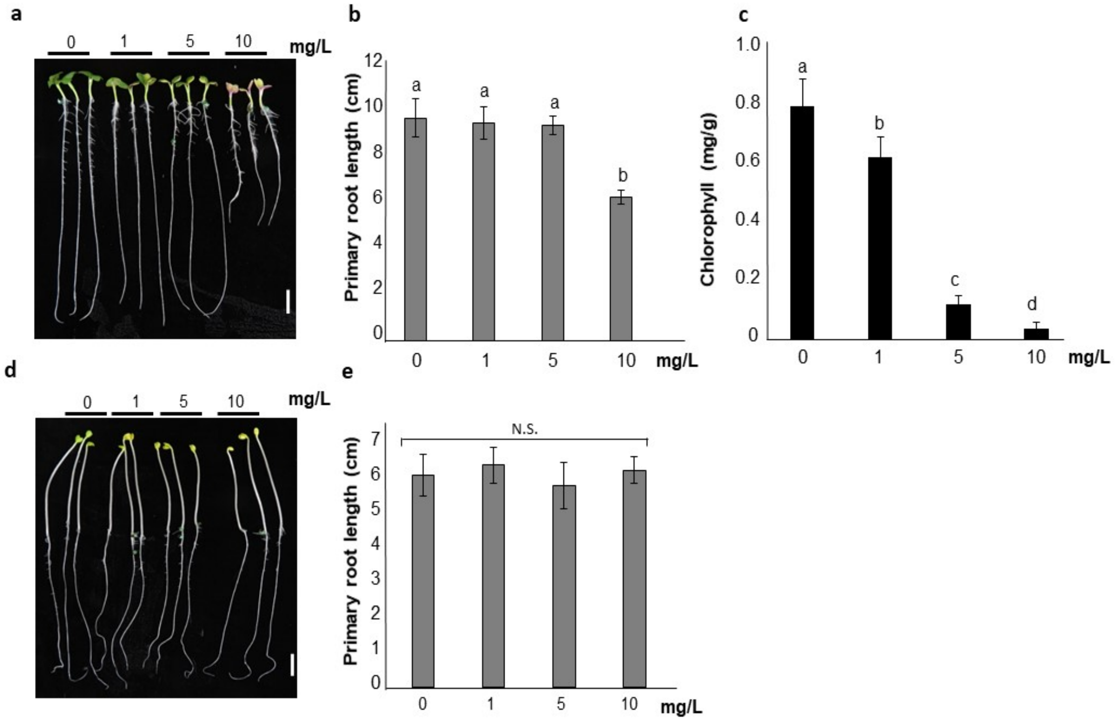

Figure S2. Comparison of the effects of different concentrations of Ery on *B. campestris* seedlings grown in long-day photoperiods and darkness. Sterilized seeds were placed on indicated Ery concentration (0, 1, 5, and 10 mg/L) -containing medium and grown vertically for four days. (**a**) Morphological phenotype at day 4 grown under long day condition (16 h light/8 h dark). (**b**) Primary root length of a. Data represent mean ± SD (*n* = 42). (**c**) Chlorophyll contents extracted from cotyledon leaves of a. Data represent mean ± SD (n=18). (d) Morphological phenotype at day 4 grown under continuous dark). (e) Primary root length of d. Data represent mean ± SD (*n* = 45). All experiments were replicated three times with similar results. Different letters above bars indicate statistically significant difference within each treatment as determined by one-way analysis of variance (ANOVA) and Turkey's honestly significant difference (HSD), p<0.05. N.D.=no significantly differences among treatments.

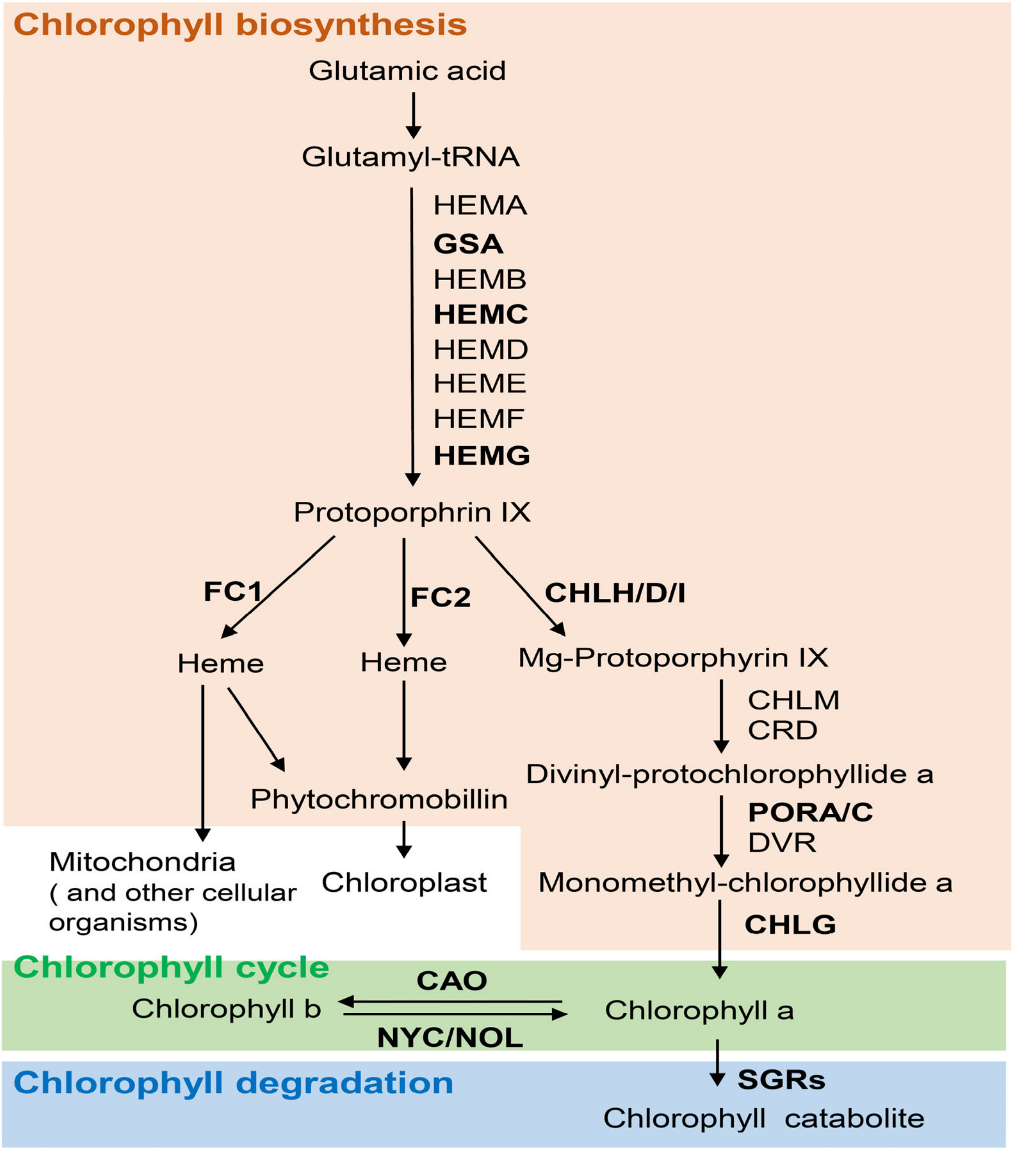

Figure S3. The chlorophyll biosynthesis pathway and the chlorophyll cycle. HEMA: glutamyl-tRNA reductase; GSA: glutamate-1-semialdehyde2,1-aminomutase; HEMB: glutamate-1-semialdehyde 2,1-aminomutase; HEMC: glutamate-1-semialdehyde 2,1-aminomutase; HEMD: uroporphyrinogen-III synthase; HEME: uroporphyrinogen decarboxylase; HEMF: coproporphyrinogen III oxidase; HEMG: oxygen-dependent protoporphyrinogen oxidase; CHLH: magnesium chelatase subunit H; CHLD: magnesium chelatase subunit D; CHLI: magnesium chelatase subunit I; FC1: ferrochelatase 1; FC2: ferrochelatase 2; CHLM: magnesium-protoporphyrin IX methyltransferase; CRD: magnesium-protoporphyrin IX monomethyl ester cyclase; PORA: protochlorophyllide reductase A; PORC: protochlorophyllide reductase C; DVR: divinyl chlorophyllide a 8-vinyl-reductase; CHLG: chlorophyll synthase; CAO: chlorophyllide a oxygenase; NYC: non-yellow coloring; NOL: non-yellow coloring-like; SGR: stay-green. Expression levels of the genes marked in bold font are shown in Figure S4 and Figure 3.

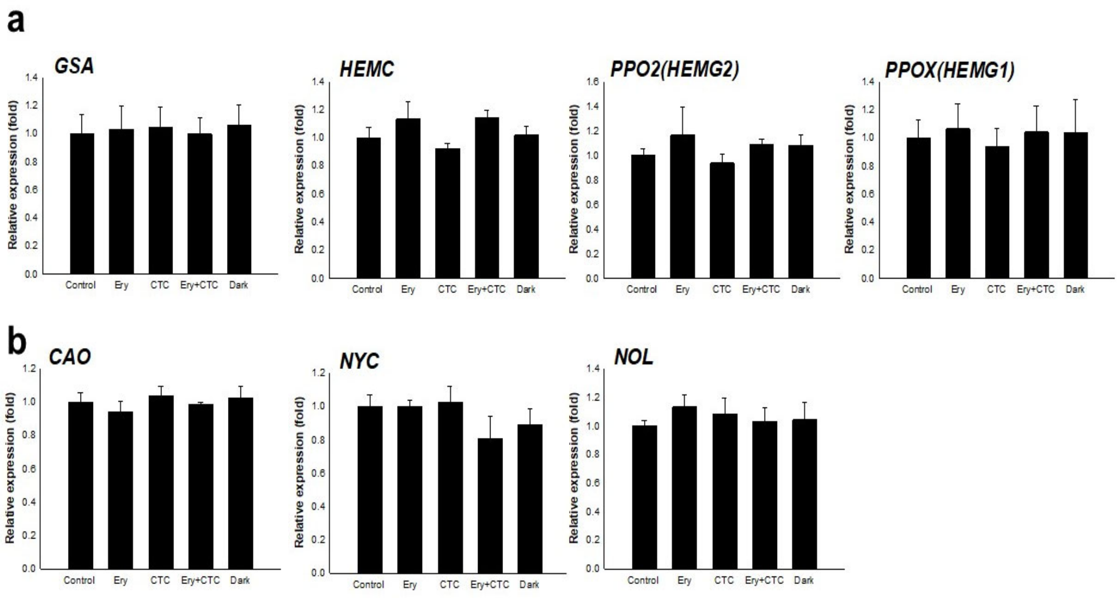

Figure S4. Effect of veterinary antibiotics on expression of genes involved in the protoporphrin IX biosynthesis and chlorophyll conversion. regulation of chlorophyll content. Expression levels of the genes marked in bold in Figure 3 were determined by qRT-PCR analysis of total RNA extracted from *B. campestris* seedlings grown in the light for 5 days under the following conditions: Control: No antibiotics; Ery: 5 mg/L erythromycin; CTC: 5 mg/L chlortetracycline; Ery+CTC: 5 mg/L erythromycin and 5 mg/L chlortetracycline; Dark: No antibiotics and complete darkness for 5 days. (a) Expression of the protoporphrin IX biosynthetic genes; *GSA* (*Bra035836* and *Bra038646*), HEMC (*Bra007749*), PPOX (*HEMG1* and *Bra033317*), and *PPO* (*HEMG2* and *Bra008772*). (b) Expression of the chlorophyll convergent genes; *CAO* (*Bra036948*), *NYC* (*Bra034882* and *Bra032697*), and *NOL* (*Bra005827*). Expression of each gene was normalized against expression of the reference gene *EF1α*. Bars represent means ± SD (*n* = 20); the experiments were replicated three times with similar results. Different letters above bars indicate statistically significant difference within each treatment as determined by one-way analysis of variance (ANOVA) and Tukey's honestly significant difference (HSD), $p<0.05$. All data are not significantly differences among treatments.

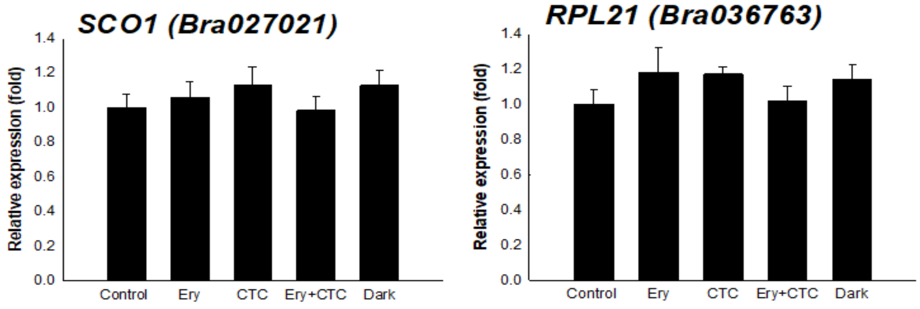

Figure S5. Effect of veterinary antibiotics on expression of *SCO1*, *RPL21*. SCO1(Bra027021, snowy cotyledons 1) and RPL21(Bra036732, Ribosomal protein L21) play roles in protein synthesis. Total RNAs were extracted from *B. campestris* seedlings treating indicated conditions. The relative transcript level of SCO1 and RPL21 was analyzed by quantitative real-time PCR (qRT-PCR). The expression of genes was normalized to that of *EF1a*. The values are means ± SD (n=12) and three-time replicates are showed similar results. Different letters above bars indicate statistically significant difference within each treatment as determined by one-way analysis of variance (ANOVA) and Turkey's honestly significant difference (HSD), $p<0.05$. All data are not significantly differences among treatments.

Table S1. List of primer sequences used in this study.

Gene	Accession	Sequence (5'→3') Forward	Reverse	
EF1a		CCCTCCGTCTACCACTTCAG	CACAACCATACCAGGCTTGA	Normalization
GSA	*Bra035836*<br>*Bra038646*	GAAGGTGTTTACTTTGCACC	GTGAGCCAAGCTAGTAAACC	Tetrapyrrole biosynthetic pathway
HEMC	*Bra007749*	GTTGCCTGCATTGGAGAGAC	TCCATGATGCTGTCGACCCA	
HEMG1	*Bra033317*	GCCTCAAGCCATTCCTCAGT	CCAGACGACGAGAGAGATGC	
HEMG2	*Bra008772*	GGGATTGAAGGTGAACCCGT	CCCGCCTCGATGATTACCTG	
FC1	*Bra009876*	AGCTAGCTTTGGAGTCAGGG	GAACCGAGCCGAAGAACATC	Fe-Chelatase
FC2	*Bra007748*	TGGTATCAGCGGGAGGGATA	CAGGTCCAACTCTGCTCTGA	
IM	*Bra013604*	AAGAAATCGCCTCCACCTGA	GCTCTCATCGTCTTGCAGTG	
CHLH	*Bra006208*	CTGGTGGTGATGGACAACGA	CCGGTCTAGCCCCAATCATC	Mg-Chelatase
CHLD	*Bra018619*	TGGATGCAACCCTTAGAGCG	CCAGCTTTCCTTGCCATTCG	
CHLI	*Bra012595*<br>*Bra013314*	TGTTGATGGGTTGAGAGGAG	CATCTGCAGTCACTCTATCTT	
PORA	*Bra003004*	GGAACAAGACCTCGGCTTCA	CCACGAGCTTCTCGCTAACT	Chlorophyll biosynthesis
PORC	*Bra033415*<br>*Bra030540*	TACCCTGGTTGCATCGCTACA	TCACTCACAACCTGTGCTAG	
CHLG1	*Bra040893*	GCTTTGGGAGGGTCCTTGTT	ATCGCTATTCCCAACCCAGC	
CHLG2	*Bra028411*	GTCCAAACCCGATTTCGCTG	GTAACTGACCCGGAAGGGAC	
CAO	*Bra036948*	GTCCTCTTGATCTCGGCTCG	AGGTCCATTACAAGCTCGGC	Chlorophyll cycle
NYC1	*Bra034882*<br>*Bra032697*	GATGACCAAGGACGGGCGTT	GTATTCTCTGTATACATCTCC	
NOL	*Bra005827*	CTCTCCCCTTGTAACGGTCG	GTCATCGGCTCTCTTGTCCC	
SGR1	*Bra019346*	TCAGTTGCAAGGATGGGCAA	CCAAAGGGGACCCCAACATT	Chlorophyll degradation
SGR2	*Bra000755*			
RBCL	*Bra028087*	TTGCCGAGATAATGGCCTAC	TCATCGCGCAGTAAATCAAC	Photosynthesis
LHCB3.1	*Bra002999*	TGAAGGCGGTTTGGACTACT	GCGAAAGTAACCGGGTCATC	
LHCB4.2	*Bra029732*	GGTTTCCGACATTAGCTCCA	TGCCTAAACCAAAAGGATCG	
PSBP-1	*Bra031534*	GATGGGTTCAAAGTGCAGGT	TTCGGGAGAACCGTAATCAG	
PETC	*Bra000837*	CTACCTTCTTTGCCCCTCCT	ATGGCACAACACATCCAAGA	
PETC	*Bra034200*	ATGCCCTTGGAAACGATGTA	TGGCACAACACATCCAAGAT	
PSAD-1	*Bra036240*	GAAGGTCCGAACTTGCTGAA	CTCCGGATAAACCCCATCTT	
SCO1	*Bra027021*	TGGAAGCGGGATCAGGATAC	AACAAGCACGGACATCAACC	Protein synthesis
PL21	*Bra036763*	ATCTCTACACTCAGCGGCTC	CTCGTATTTGGCTGTCGGTG	
RBOHD	*Bra037520*<br>*Bra020724*	TGGGTGACTAGGGAACAAGG	CATCAACACCGTTCTTAGCG	Oxidative stress
RBOHF	*Bra027764*	GAACAGCACAGGAAGCAACA	GCATCTCTATCACACCCCGT	
FSD1	*Bra013863*	ACTCCCAATGCTGTGAATCC	GGCACTTACAGCTTCCCAAG	

FSD2	*Bra029190*<br>*Bra035724*	CCAACGGAGGAAGACAAGAA	GTTTCCCATGACACGAGCTT
FSD3	*Bra026503*<br>*Bra009670*	GGATGTGTGGGAGCACTCTT	GATTGGGATGTTGGGTTCAC
CSD3	*Bra002133*	ACGGTGTTGCTGAAATCT	CCGCAGATGATTGAAGTCCG
